# Mediastinal hemangioma mimicking an invasive tumor growth: A case report

**DOI:** 10.1016/j.ijscr.2021.105674

**Published:** 2021-02-23

**Authors:** Shogo Yobita, Shuhei Iizuka, Yoshiro Otsuki, Toru Nakamura

**Affiliations:** Departments of General Thoracic Surgery and Pathology, Seirei Hamamatsu General Hospital, 2-12-12, Sumiyoshi, Nakaku, Hamamatsu-city, Shizuoka, 430-8558, Japan

**Keywords:** CT, computed tomography, MRI, magnet resonance imaging, Case report, Mediastinal tumor, Hemangioma, Dynamic CT

## Abstract

**Introduction and importance:**

Mediastinal hemangiomas are a rare entity frequently developing in the anterior mediastinum. They may exhibit an infiltrating appearance into the surrounding tissue, and extensive surgery is often required despite its benign nature. We report a case of a mediastinal hemangioma mimicking an invasive tumor growth requiring a combined resection of the lung and diaphragm.

**Case presentation:**

An asymptomatic 73 year-old-man presented with a 50 mm-sized mass on his chest radiography. A combined resection of the mediastinal mass with the part of the lower lobe of left lung and diaphragm not necessitating a patch repair was performed. The pathological findings were compatible with a diagnosis of a mediastinal hemangioma. Only fibrous adhesions were observed between the tumor and resected lung and diaphragm without any histological invasion.

**Clinical discussion:**

While imaging examinations play a key role in determining a preoperative diagnosis, mediastinal hemangiomas may pose a diagnostic challenge mainly due to its rarity. With a clinical suspicion of a hemangioma during the diagnostic work up, a dynamic CT might be helpful.

**Conclusions:**

In mediastinal hemangiomas, a preoperative diagnosis is essential in order to avoid extensive surgery. A hemangioma should be raised as a differential diagnosis for anterior mediastinal tumors, especially in cases with an infiltrative appearance that suggests the necessity for a combined resection of the surrounding organs.

## Highlights

• Mediastinal hemangiomas may exhibit an infiltrating appearance.
• Extensive surgery is often required due to an inaccurate preoperative diagnosis.
• A hemangioma should be raised as a differential diagnosis to avoid extensive surgery.

## 1 Introduction

Mediastinal hemangiomas are rare neoplasms accounting for 0.5% of mediastinal tumors without any specific imaging findings [1,2]. They may exhibit an infiltrating appearance into the surrounding tissue, and extensive surgery is often required despite its benign nature [3,4]. We herein report a case of a mediastinal hemangioma mimicking an invasive tumor growth that required a combined resection of the lung and diaphragm. This work has been reported in line with the SCARE criteria [5].

## 2 Case presentation

**Fig. 1:**
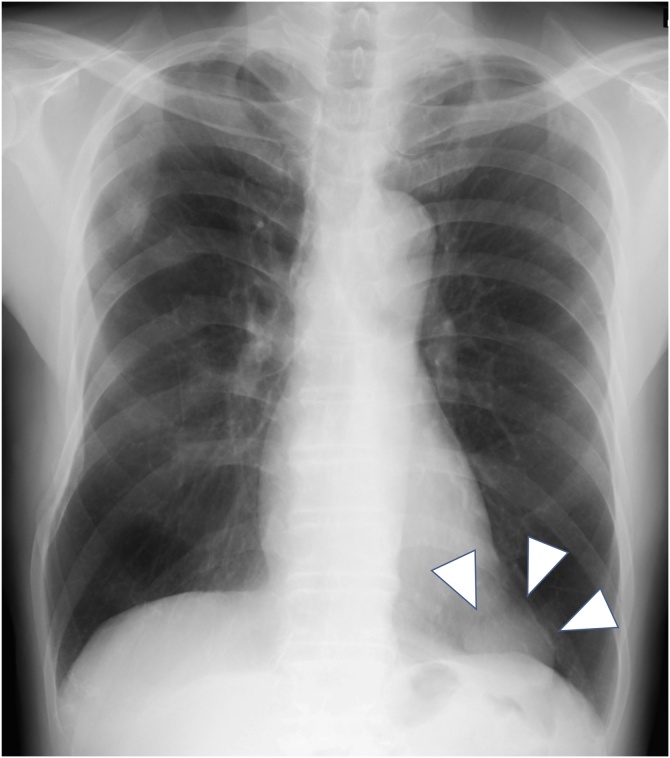
Chest X-ray showing a 50 mm-sized mass in the lower left lung field (arrowheads).

**Fig. 2:**
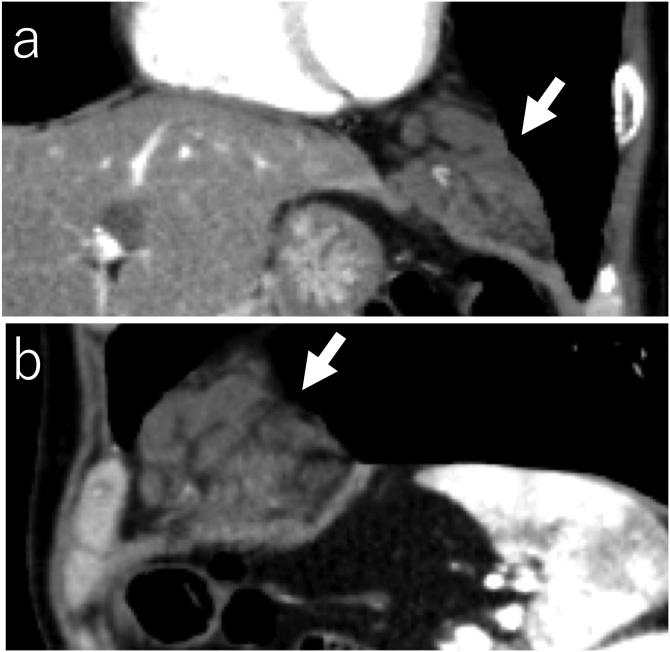
a) Chest contrast-enhanced computed tomography showing an irregular mass (arrows) with a maximum diameter of 55 mm in the anterior mediastinum, which extends along the left diaphragm (a: coronal plane, b: sagittal plane).

**Fig. 3:**
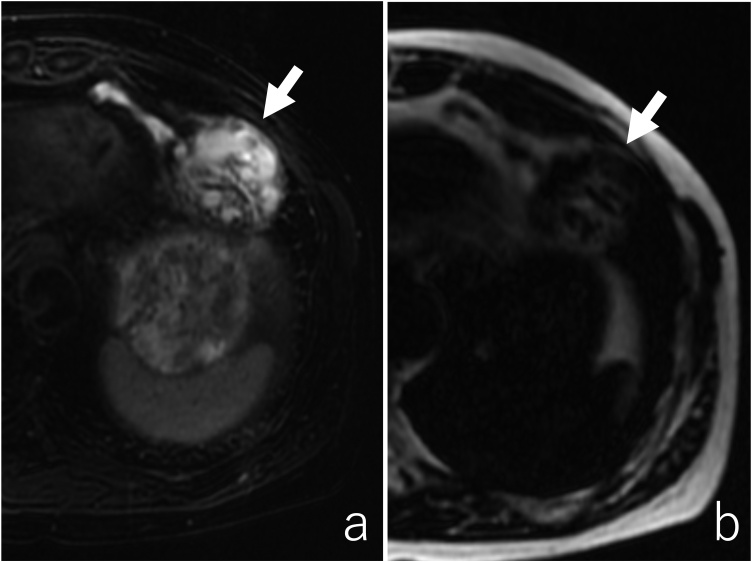
a) Magnetic Resonance Imaging revealed a septate cystic lesion with a high intensity on the T2-weighted image. b) Fat-suppressed T1-weighted images revealed that the mass contained less fatty tissue (arrows).

An asymptomatic 73 year-old man with a remote history of surgically treated gastric cancer at the age of 40 presented with a 50 mm-sized mass on a chest radiography (Fig. 1). He had no significant familial history without any drug use nor allergic history. Chest contrast-enhanced Computed Tomography (CT) revealed an irregular homogenous mass in the anterior mediastinum, which extended along the left diaphragm without any specific enhancing effects (19–27 Hounsfield Unit) (Fig. 2ab). Magnetic Resonance Imaging (MRI) revealed a septate cystic lesion with a high intensity on the T2-weighted image (Fig. 3a). Fat-suppressed T1-weighted images revealed that the mass contained less fatty tissue (Fig. 3b). An invasive thymic epithelial tumor or soft tissue tumor were suspected mainly because of its location and a radiological extent, and a radical excision was planned via video assisted thoracoscopic surgery by an attending thoracic surgeon at our institute.

**Fig. 4:**
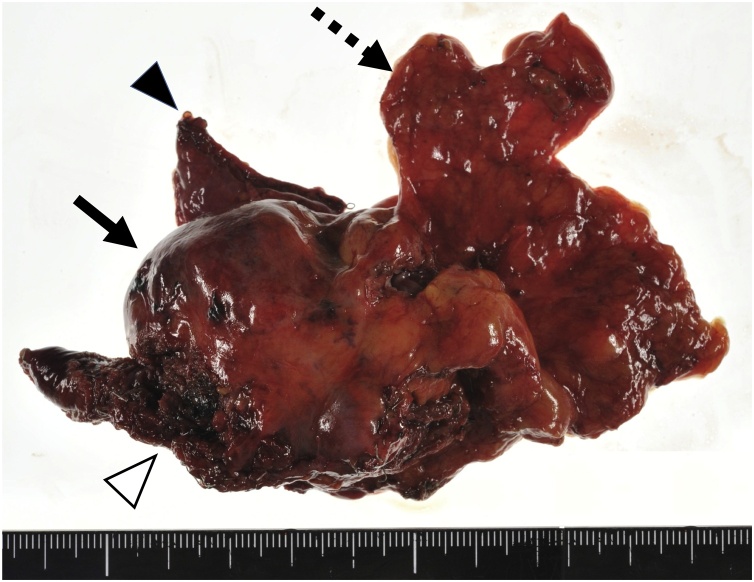
Gross findings of the surgical specimen showing the circumscribed mass (arrow) with the pericardial fat (dotted arrow), left lung (arrow head), and diaphragm (white arrow head).

**Fig. 5:**
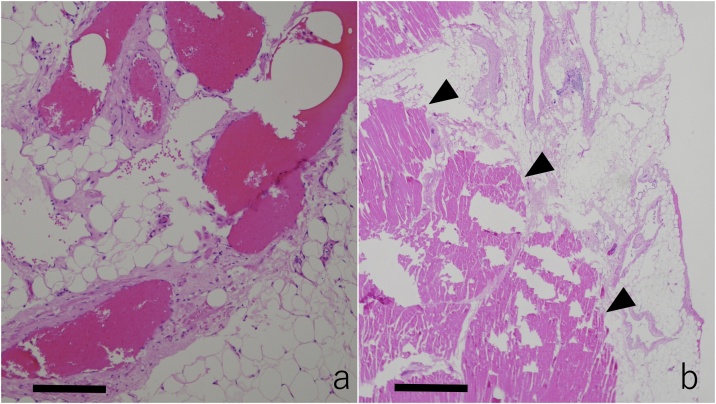
a) The histopathological findings showing dilated medium sized blood vessel proliferation in the adipose tissue (arrows, the scale indicates 200 μm). Those vessels were mainly muscular veins with a small number of arteries. b) Only fibrous adhesions were observed between the tumor and resected diaphragm (arrowheads, the scale indicates 1000 μm).

With the patient in the right semi lateral decubitus position, a total of 3 ports were placed on the 6th, 7th, and 8th left intercostal anterior axillary lines, respectively. Under Carbon dioxide insufflation at a pressure of 8 mmHg, the lesion was visualized adjacent to the pericardium. The lesion was also observed to have grossly invaded into the diaphragm and lower lobe of the left lung. He underwent a total tumorectomy with a combined resection of the pericardial fat, left lung, and diaphragm. Since a complete resection was achieved, no intraoperative frozen section examination was performed. The postoperative course was uneventful and the patient was discharged on the third postoperative day. The macroscopic findings of the specimen revealed a circumscribed mass embedded in the fat tissue (Fig. 4). The histopathological findings revealed dilated medium sized blood vessel proliferation in the adipose tissue. Those vessels were mainly muscular veins with a small number of arteries. Those findings were compatible with the diagnosis of a mediastinal hemangioma (Fig. 5a). Only fibrous adhesions were observed between the tumor and resected lung and diaphragm without any histological invasion (Fig. 5b).

## 3 Discussion

Mediastinal hemangiomas are rare neoplasms accounting for 0.5% of mediastinal tumors and are frequently found in the anterior mediastinum [1,2]．They have no specific symptoms and may present with a variety of clinical manifestations such as a cough, respiratory distress, and chest pain [2,4]. They often require surgical intervention to avoid disease related morbidities such as massive hemoptysis or respiratory failure [6,7].

While most cases are generally visualized with well circumscribed masses [4,8,9], some can exhibit a wide range of radiological findings mimicking dumbbell tumors [3,10] or an infiltrative-like progression as shown in the present case [11]. Unlike other mediastinal tumors, even an incomplete resection would be feasible for hemangiomas without a local recurrence, malignant degeneration, hemorrhagic morbidity, or becoming symptomatic [1,9]. Therefore, a preoperative diagnosis is essential to avoid extensive surgery. While imaging examinations play a key role, mediastinal hemangiomas may pose a diagnostic challenge mainly due to its rarity. Positron emission tomography CT was not available in the present case and its diagnostic significance in hemangiomas is unclear [12].

On MRI, a fat component and high intensity on the T2-weighted images have been reported as characteristic of a mediastinal hemangioma and likewise of hepatic hemangiomas [12,13]. Part of those findings were detected in the present case but were not diagnostic because a hemangioma had not been raised as a differential diagnosis due to its rare frequency. Peripheral nodular enhancement on the dynamic MRI has been also reported to be diagnostic but was not performed in this case [[14], [15], [16]].

Calcified phleboliths derived from organized thrombi are the most diagnostic sign, and are detected more sensitively by CT than conventional radiography [12,17]. However, they are not so frequent and account for only 10–52.9% at most [2,13,18,19] and were not identified in this case.

On the other hand, it has been reported that peripheral puddle images showing a punctate contrast effect on the margin of the tumor in the aortic phase on the dynamic CT is highly specific for hepatic hemangiomas [20], and its usefulness has also been reported for mediastinal hemangiomas with a sensitivity of about 70% [[14], [15], [16]]. With a clinical suspicion of a hemangioma during the diagnostic work up, a dynamic CT might have been helpful and we could have chosen a more limited surgery and avoided the extensive resections in this case.

## 4 Conclusions

Mediastinal hemangiomas are a rare entity frequently developing in the anterior mediastinum. They could exhibit an infiltrating appearance and often are amenable to extensive surgery despite being totally benign in nature. Therefore, a preoperative diagnosis is essential to avoid extensive surgery. A hemangioma should be raised as a differential diagnosis of anterior mediastinal tumors, especially in cases with an infiltrative appearance suggesting the necessity for a combined resection of the surrounding organs.

## Declaration of Competing Interest

The authors report no declarations of interest.

## Funding

Not applicable.

## Ethical approval

Not applicable.

## Consent

Written informed consent was obtained from the patient for publication of this case report and accompanying images. A copy of the written consent is available for review by the Editor-in-Chief of this journal on request.

## Author contribution

Shogo Yobita wrote this paper. All authors read and approved the final manuscript.

## Registration of research studies

Not applicable.

## Guarantor

Toru Nakamura.

## Provenance and peer review

Not commissioned, externally peer-reviewed.

## Availability of data and material

Not applicable.
